# Evaluation of Intracranial Arteriovenous Malformations Using Ischemic Stroke Color-Coded Maps Software, a New Rapid Post-Processing Tool in CT Angiography

**DOI:** 10.3390/jcm14165833

**Published:** 2025-08-18

**Authors:** Francesco D’Argento, Tommaso Verdolotti, Rosa D’Abronzo, Davide De Leoni, Emanuele Ferravante, Francesco Arbia, Marta Iacobucci, Simona Gaudino, Matteo Mancino, Chiara Schiarelli, Giuseppe Garignano, Alessandro Pedicelli

**Affiliations:** 1 UOSA Neuroradiologia Interventistica, Fondazione Policlinico Universitario “A. Gemelli” IRCCS, 00168 Rome, Italy; giuseppe.garignano@policlinicogemelli.it (G.G.); alessandro.pedicelli@policlinicogemelli.it (A.P.); 2 UOSA Neuroradiologia Diagnostica, Fondazione Policlinico Universitario “A. Gemelli” IRCSS, 00168 Rome, Italy; tommaso.verdolotti@policlinicogemelli.it (T.V.); simona.gaudino@policlinicogemelli.it (S.G.); chiara.schiarelli@policlinicogemelli.it (C.S.); 3 Dipartimento di Diagnostica per Immagini, Radioterapia Oncologica ed Ematologia-Istituto di Radiologia, Fondazione Policlinico Universitario “A. Gemelli” IRCCS, 00168 Rome, Italy; rosadabronzo@hotmail.it (R.D.); deleonidavide3@gmail.com (D.D.L.); emanuele.ferravante@gmail.com (E.F.); matteomancino@gmail.com (M.M.); 4 NESMOS, Department of Neuroradiology, S. Andrea Hospital, University Sapienza, 00168 Rome, Italy; francescoarbia@hotmail.it; 5 Neuroradiology Unit, University Hospital Policlinico Umberto I, Department of Human Neurosciences, Sapienza University of Rome, 00161 Rome, Italy; marta.iacobucci@gmail.com

**Keywords:** stroke, arteriovenous malformations, CT angiography, digital subtraction angiography, color-coded maps

## Abstract

**Background/Objectives**: In patients with intracranial arteriovenous malformation (AVM), the first diagnostic analysis is often performed in emergency conditions by Computed Tomography (CT) and multiphase CT angiography (CTA). Nevertheless, once ruptured, an AVM might be hardly recognized by an inexperienced neuroradiologist, due to the presence of hematoma or to the destructuring of the lesion. The aim of our study is to outline the utility of color-coded maps derived from multiphase CT angiography in the assessment of cerebral AVMs, evaluating inter-observer agreement between radiologists with different years’ experience and comparing the results with the gold standard, angiography. **Methods**: The color-coded maps were obtained retrospectively by multiphase CT angiography on a workstation using FastStroke software ColorViz (GE Healthcare, Milwaukee, WI, USA). The color-coded maps were evaluated, independently, by two neuroradiologists, and inter-observer agreement was evaluated. Finally, the AVM’s features (arterial feeders, nidus type, venous drainage type) obtained with color-coded maps were compared with angiographic analysis. The Ethical Committee for Research in Medical Imaging approved this study (Institutional Review Board number 6467). Informed consent was obtained for every patient. **Results**: A total of 26 patients with intracranial hemorrhage and arteriovenous malformation underwent multiphase CT angiography and were analyzed. Our statistical analysis showed the reproducibility of the color-coded maps and agreement with the angiographic findings, especially in the evaluation of venous drainage type. **Conclusions**: The ColorViz color-coded maps have proved to be an effective tool in the identification and assessment of AVMs, providing rapid and clear information on intracranial vascular dynamics, even for inexperienced radiologists.

## 1. Introduction

**Figure 1:**
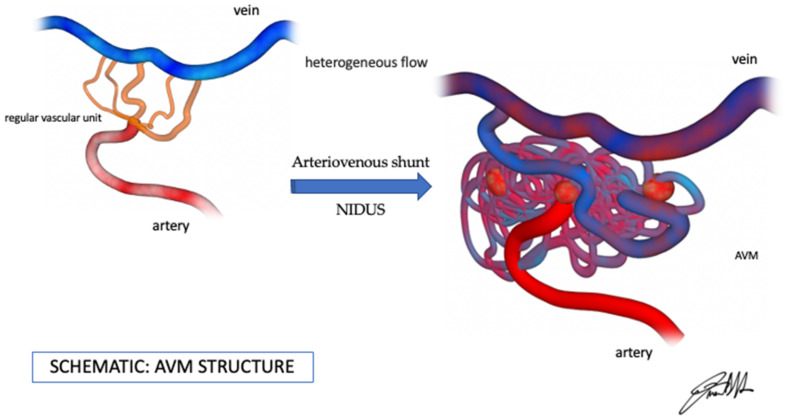
Original picture (by Dr. Francesco D’Argento), AVM hemodynamics features.

An arteriovenous shunt is an abnormal communication between an arterial and a venous channel, without the physiological presence of a normal interposed capillary network (Figure 1).

This pathological vascular structure is present in some cerebrovascular malformations, such as arteriovenous fistulas and arteriovenous malformations, conditions that may cause an intracerebral hemorrhage (ICH). Cerebral arteriovenous malformations (AVMs) are relatively rare vascular anomalies, with an estimated prevalence of approximately 10–18 per 100,000 individuals and an annual hemorrhage risk of 2–4%, making them a significant cause of ICH, particularly in younger adults [1,2].

Among these, cerebral arteriovenous malformations are characterized by a complex tangle of dysplastic vessels known as the nidus, which directly connects the arterial and venous systems without the normal capillary bed. AVMs are a significant cause of spontaneous intracerebral hemorrhage, particularly in young adults, and carry substantial morbidity and mortality risks [1,2].

Digital Subtraction Angiography (DSA) is the gold standard exam for diagnosis and characterization of cerebral AVM, due to its high spatial and temporal resolution [3].

However, since almost 50% of AVMs present with intracranial hemorrhage, the first evaluation of the patient is often performed in emergency conditions by Computed Tomography (CT) and multiphase CT angiography (MCTA) [4]. AVM can be recognized by the correct identification of nidus, appearing as a tangle of vessels and of early venous drainage, which typically enhances in the “arterial” phase as other arterial vessels. Nevertheless, once ruptured, an AVM might be hardly recognized by an inexperienced neuroradiologist, due to the presence of hematoma or to the destructuring of the lesion [5]. Recent studies have shown that AVMs may be under-recognized or misdiagnosed in emergency settings, especially by non-specialist radiologists, with error rates that may impact clinical outcomes [4,5].

Over the last years, some authors have already highlighted the usefulness and validity of post-processing software in the identification of cerebral vascular pathologies [6,7,8].

**Figure 2:**
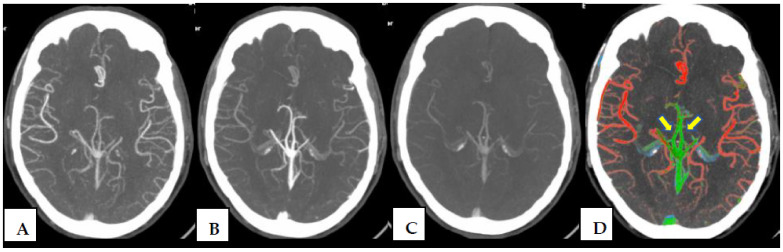
MCTA phases (**A**–**C**) and corresponding ColorViz map on a single slice (**D**). The ColorViz color-coded map allows for easy identification of both arteries (in red) and veins (in green). Specifically, in this single slice, branches of ACA (anterior cerebral artery), MCA (middle cerebral artery) and, partially, PCA (posterior cerebral artery) are instantly recognized in red; internal cerebral veins (yellow arrows) and a portion of the great cerebral vein of Galeno, the rectus sinus, and the superior sagittal sinus are displayed in green.

However, while color-coded tools like ColorViz have been validated in stroke imaging, their application to AVM assessment has not been widely explored, despite both conditions involving complex vascular dynamics. The ability of ColorViz to detect arterialized venous structures or enhancement asymmetries may be particularly beneficial in AVMs (Figure 2).

The aim of our study is to outline the utility of color-coded maps derived from MCTA in the assessment of cerebral AVMs, evaluating inter-observer agreement between radiologists with different years’ experience and comparing the results with the gold standard, DSA.

## 2. Materials and Methods

### 2.1. Study Design

From 1 January 2017 to 31 December 2022, we retrospectively selected patients in a consecutive manner. Patients who underwent multiphase CT angiography (including a basal CT) in our Radiology Department and were finally diagnosed with cerebral AVM at discharge were included. Exclusion criteria were the presence of other vascular abnormalities (such as cerebral arterial occlusion) and the presence of motion artifacts on the CT scan and, therefore, on the color-coded map.

All imaging and clinical data were created during routine clinical workup. Institutional Review Board approval was obtained. With written consent, patients or proxies were systematically informed that they could oppose the use of their data for research purposes.

### 2.2. CT Protocol and Post-Processing Tool

All subjects underwent MCTA, including basal CT, performed using a 64-multislice CT (GE MEDICAL SYSTEM Optima CT660 645, GE Healthcare, Milwaukee, WI, USA). After a basal CT scan was acquired and 80 mL of contrast media at 4 mL/s flow was injected, the acquisition was composed of three subsequent phases (separated by an interval of 8 s): an arterial phase extending from the aortic arch to the vertex, and two following phases (acquired in the early and late venous phases) from the occipital foramen to the vertex.

The color-coded maps were obtained on a workstation using FastStroke (version 1.0.) software (GE Healthcare, Milwaukee, WI, USA) that automatically elaborates the full set of images included in the CT protocol (basal CT and MCTA). As mentioned above, on the color-coded map, the vessel shows different color (red, green and blue) depending on the arrival time of the contrast medium and on a per-person adaptive threshold technique: briefly, red for vessels that enhance maximally in arterial phase, and green and blue for vessels that enhance maximally in early venous phase and late venous phase, respectively.

### 2.3. DSA (Digital Subtraction Angiography)

DSA imaging was performed for 21 patients by an experienced interventional neuroradiologist (F.D. with 15 years’ experience) on a Biplane Angiography unit (Siemens, Munich, Germany), after femoral or radial puncture in local or general anesthesia (based on the clinical condition of the patient), by selective catheterization and contrast injection of the supra-aortic trunks at 3 and at 6 frames/s in standard orthogonal anteroposterior, lateral, and oblique projections. The study was completed with 3D rotational angiography.

### 2.4. Image Interpretation and Statistical Analysis

The color-coded maps were evaluated, independently, by two neuroradiologists (T.V., the first reader, and R.D., the second reader) with 15 years’ and 4 years’ experience, respectively. Each one assessed the presence and number of vascular malformations and evaluated nidus, arterial feeder, and venous drainage. Specifically, for each AVM recognized, each operator evaluated the presence of nidus (yes/no), nidus type (compact/diffuse), arterial feeder (single/multiple), venous drainage (superficial veins/deep veins/both), and cerebral venous sinus color asymmetry (yes/no) (Table 1).

Hence, inter-observer agreement was evaluated using Cohen’s kappa coefficient, which goes from “no agreement” (K < 0) to “almost perfect agreement” (K > 0.8) [9].

Finally, for 21 patients with AVM, the evaluation of nidus type, arterial feeder, venous sinus color asymmetry, and venous drainage type performed by both color-coded maps neuroradiology readers (T.V. and R.D.) was compared to angiographic findings by the neurointerventional operator (F.D.).

Agreement on each AVM component for each reader pair was determined using weighted k, a measure of agreement ranging from 0 to 1 that takes into consideration the odds of agreement due to chance [10]. Given the relatively small sample size, the interpretation of Cohen’s kappa values should be approached cautiously, as small sample sizes can result in wide confidence intervals or unstable estimates.

K values were determined for agreement across all AVM components separately.

No *p*-values were reported due to the limited sample size, which precludes reliable statistical significance testing.

Statistical analysis was performed using GraphPad Prism version 9.0.0 for Mac, GraphPad Software, San Diego, CA, USA, www.graphpad.com.

## 3. Results

We selected 30 subjects from our institutional database. According to the aforementioned criteria, four were excluded due to a combination of other vascular abnormalities (one had additionally an occlusion of an internal carotid artery) and due to technical issues. Finally, 26 patients were included, whose characteristics are reported in Table 2.

Nineteen of the patients presented with cerebral hemorrhage. The other seven patients with unruptured AVM have presented for symptoms, in particular, three for epileptic seizure, two for headaches, one for syncope and disorientation, and one for ischemic stroke due to occlusion of a sylvian branch.

Out of these 26 patients, 21 additionally underwent angiography

Inter-observer agreement between the two neuroradiologists’ (Table 3) and the neurointerventional radiologist’s (Table 4 and Table 5) readings was expressed in percentage observed agreement for each AVM component and by calculating Cohen’s kappa, both with corresponding 95% confidence intervals.

For the “nidus presence” readings, calculated only between the expert and non-neuroradiologists, the two readers reached a good agreement (K = 0.621) with a proportion of agreement in 92% of the observations. Furthermore, the colorimetric maps allowed the identification of “venous drainage type” and evidence of “flow arterialization” in efferent venous structures, with moderate agreement between the two observers with different experience (respectively, K = 0.567 and K = 0.434).

The results showed agreement with the angiographic findings. However, agreement was lower for features such as nidus type and arterial feeder, possibly due to ambiguous operational definitions or differing interpretations of compact vs. diffuse patterns, especially without standardized training between readers. Specifically, in fact, regarding the type of venous drainage, both observers (neuroradiologists) who used ColorViz reached an agreement with K > 0.746 (“substantial agreement”) compared with the angiographic detection made by the third operator.

In comparison, moderate agreement has been reported between the second reader and the neurointerventional radiologist on the “nidus type” voice, but no agreement with the first reader.

**Figure 3:**
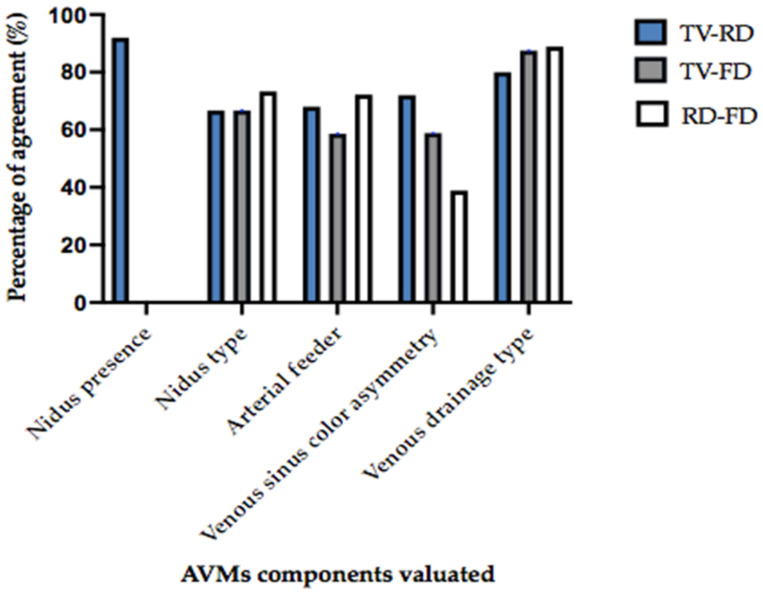
Histogram of the percentage of agreement (Y-axis) in evaluating the different AVM components (X-axis) between different operators. The “nidus presence” has been evaluated between the expert neuroradiologist and non-expert neuroradiologist (“TV”, ”RD”) only. All the other components have been evaluated by the two neuroradiologists and the angiographer (FD). The highest percentage of agreement (from 80% to 88,9% in percentage, and Cohen’s K from 0.56 to 0.75) was found in evaluation of the venous drainage type between all three operators.

The highest percentage of agreement between all three operators was found in the evaluation of venous drainage type (from 80% to 88,9% in percentage, and Cohen’s K from 0.56 to 0.75) (Figure 3).

## 4. Discussion

The characterization of the AVM (Figure 1) is important to predict its risk of hemorrhage and comprises the description of the arterial feeder, the nidus, and the draining vein. Cerebral AVMs may sometimes present with stroke-like symptoms or may be an incidental finding on CT angiography.

The digital subtraction angiography (DSA) is the gold standard exam for cerebrovascular diseases, and so for the characterization of the cerebral AVM. The main DSA advantages in the evaluation of AVMs lie in its dynamicity, in its highest spatial resolution (0.2 mm) and temporal resolution (up to 24 frames per second) [11,12,13,14].

These features help to detect an early draining vein relative to the rest of the normal draining venous system of the parenchyma and to identify small nidus, occasionally not visible on CT and RM scans. It also provides information on hemodynamic features (high or low flow shunt) and angioarchitecture, it helps in rupture prediction of the AVM, and it is important for therapeutic decisions (follow-up, endovascular treatment, surgical treatment, or radiosurgical therapy) [15]. 

Nevertheless, DSA is not a totally risk-free modality, considering contrast agent injection risks (i.e., allergy and nephrotoxicity), radiation exposure, and procedural risks; additionally, it is not available in all centers [16,17].

Hence, several studies have also assessed the potential utility of other non-invasive image modalities, such as Magnetic Resonance Imaging (MRI). Among MRI angiographic sequences, 4D-MRA and 4D flow MRI (which adds a new dimension: quantitative three-directional blood flow velocities), with both high spatial and high temporal resolution, have been emerging in the evaluation of intracranial vascular dynamics and, specifically, in AVM evaluation, before and after treatment [18,19,20,21].

However, although vascular malformations, AVMs’ most common presentation is intracranial bleeding [1,5]. Hence, the first evaluation of AVM is often performed in emergency conditions, when the malformation is already ruptured, resulting in intraparenchymal hemorrhage (IPH), intraventricular hemorrhage (IVH), or subarachnoid hemorrhage (SAH) [4].

In the emergency settings, CT is the modality of choice and AVM bleeding, since atypical, is generally assessed with both basal CT and MCTA. The correct identification of this kind of malformation relies on the identification of nidus and of early venous drainage, which can be made only if veins are enhanced in the “arterial” phase [5]. Yet, this might be difficult to recognize, especially by an inexperienced neuroradiologist, due to the presence of a hematoma or the destruction of the lesion. Furthermore, the assessment should be performed in short times, considering the urgency of these conditions and their possible complications.

ColorViz is a post-processing tool that combines data from basal CT and MCTA, summing in a single color-coded view the different CTA phases. It provides rapid and clear information about intracranial vascular dynamic assigning a different color to each vessel (Figure 2) according to its density changes over time; specifically, the vessels colors can be red (those that enhance maximally during the arterial phase), green (those that enhance in the early venous phase), and blue (those that enhance in the late venous phase). To date, this tool has only been assessed in patients with stroke, despite its potential utility in other vascular intracranial abnormalities [6,8].

In our experience, the color-coded maps represent a valuable support tool in the evaluation of AVMs, with good agreement between operators with different years of experience (15 years and 4 years) and short post-processing times, extremely helpful in emergency conditions. It also provides insight into the angioarchitecture of the vascular malformation, allowing a rapid and clear identification of each AVM component (arterial feeder, nidus, venous drainage).

In our analysis, the AVMs are all recognized by both operators. Mostly, the nidus appears as a tangle of red vessels, connected to arterial feeders and venous drainage, which generally also presents as the same color vessels, due to the “arterialization” of the vein. This appearance facilitates the identification of the malformation and, especially, the venous drainage, both superficial and deep. This is particularly easy for bilateral venous structure (i.e., internal cerebral veins or bilateral dural sinus) since the one affected shows a different color from the other (normal) one; hence, asymmetry of venous structures’ color is extremely helpful in the identification of venous drainage.

Although venous drainage type demonstrated substantial inter-observer agreement, the lower concordance for nidus type and arterial feeders likely reflects their greater interpretative complexity. This highlights the importance of more precise definitions and the potential value of quantitative tools to enhance consistency in future evaluations.

Finally, results were compared to those of angiography, showing promising results, especially in the evaluation of venous drainage type, with an agreement above 85% for both operators. Interestingly, the junior neuroradiologist demonstrated slightly better agreement with angiographic findings for certain features. This may suggest a more impartial interpretation or a greater reliance on the objective visual cues provided by ColorViz, rather than on experience-based pattern recognition or subjective judgment. These findings highlight the potential utility of ColorViz in supporting diagnostic consistency among less experienced readers.

The post-processing time is very fast, about 10–15 s, and especially the diagnostic times appear overall faster in ColorViz than in MCTA alone. This aspect could be very important in an emergency context.

Obviously, the main limitation of this study is the relatively small sample size and its retrospective design. These factors may affect the generalizability and statistical power of our findings, particularly in the interpretation of subtle variations in AVM imaging features. The limited number of patients, especially those who underwent both CTA and DSA, may also contribute to the wide confidence intervals observed in some interrater reliability measures, and may not capture the full spectrum of AVM angioarchitectures.

Furthermore, as this was an exploratory study, no a priori power analysis was performed, and the imaging assessments were based on visual interpretation rather than standardized quantitative thresholds. These aspects introduce potential bias and subjectivity, despite the encouraging inter-observer agreement observed.

Another limitation is the lack of sensitivity and specificity analyses comparing ColorViz-based CTA assessments with DSA. This aspect should be addressed in future studies.

Future prospective studies with larger and more diverse patient populations are needed to validate our preliminary observations. Such studies could also provide deeper insight into the diagnostic utility of ColorViz, help to identify potential AVM subtypes based on distinct enhancement patterns, and explore the clinical significance of color-coded alterations in AVM components.

## 5. Conclusions

In emergency settings, cerebral AVM diagnosis represents a tough challenge for neuroradiologists, especially considering the coexistence/overlap of intracranial bleedings, the lack of patients’ collaboration, and the short time available for an adequate evaluation. The MCTA is an extremely valuable modality, which, in experienced hands, allows one to suspect AVM diagnosis and to assess its components.

The ColorViz color-coded maps have proved to be an effective tool in the identification and assessment of AVMs, providing rapid and clear information on intracranial vascular dynamics, even for inexperienced radiologists.

Our results confirm the reproducibility of the color-coded maps, evaluated by operators with different years’ experience, showing good inter-observer agreement and correlation with the modality of choice of intracranial vascular abnormalities, the DSA.

## Figures and Tables

**Table 1:** Assessment of AVM components.

ColorViz—Assessment of AVM Components	
Nidus Presence	Yes/No
Nidus type	Compact/Diffuse
Arterial feeder	Single/Multiple
Venous sinus color asymmetry	Yes/No
Venous drainage	Superficial veins/Deep veins/Both

**Table 2:** General criteria. * Age values are indicated as average; standard deviation is shown in brackets.

General Criteria		
	ColorViz	Angiography
**Patients (*n*)**	**26 (14 M; 12 F)**	**21 (12 M; 9 F)**
Age (y.o) *	**50 (±21)**	**42 (±20)**
**AVM components evaluated**	**5**	**4**
**AVM site:**		
**Supratentorial**	**24 (92%)**	**20 (95.3%)**
**Infratentorial**	**2 (8%)**	**1 (4.7%)**
	- **19: intracranial hemorrhage (AVM ruptured)** - **7: neurological symptoms (AVM unruptured)**	
**Clinical presentation**		


**Table 3:** **Color-coded map readers’ agreement.** Proportion of agreement (%), K statistics, and 95% confidence intervals for the two raters, neuroradiologists with different years of experience: T.V. (15 yrs) and R.D. (4 yrs) using ColorViz.

Color-Coded Map Readers’ Agreement			
Variable	Proportion of Agreement (*% of the Observations*)	K Value	95% CI
**Nidus presence**	**92%**	**0.621**	**0.141–1.000**
**Nidus type**	**66.67%**	**−0.089**	**−0.316**
**Arterial feeder**	**68%**	**0.123**	**−0.835**
**Venous sinus color asymmetry**	**72%**	**0.434**	**0.089–0.778**
**Venous drainage type**	**80%**	**0.567**	**0.232–0.903**

**Table 4:** **First color-coded map reader.** Proportion of agreement (%), K statistics, and 95% confidence intervals for the two raters, a neuroradiologist with 15 years of experience, T.V., and an interventional neuroradiologist, F.D.

First Color-Coded Map Reader—Neurointerventional Radiologist (Angiography) Agreement			
Variable	Proportion of Agreement (*% of the Observations*)	K Value	95% CI
**Nidus type**	**66.67%**	**−0.119**	**−0.4**
**Arterial feeder**	**58.62%**	**0.144**	**−0.756**
**Venous sinus color asymmetry**	**58.82%**	**0**	**-**
**Venous drainage type**	**87.50%**	**0.758**	**0.468–1**

**Table 5:** Proportion of agreement (%), K statistics, and 95% confidence intervals for the two raters, a neuroradiologist with 4 years of experience, R.D., and an interventional neuroradiologist, F.D.

Second Color-Coded Map Reader—Neurointerventional Radiologist (Angiography) Agreement			
Variable	Proportion of Agreement (*% of the Observations*)	K Value	95% CI
**Nidus type**	**73.33%**	0.412	−0.926
**Arterial feeder**	**72.22%**	0.444	−0.889
**Venous sinus color asymmetry**	**38.89%**	0	-
**Venous drainage type**	**88.89%**	0.746	0.424–1.000

## Data Availability

Data is contained within the article. The original contributions presented in this study are included in the article. Further inquiries can be directed to the corresponding author.

## Abbreviations

The following abbreviations are used in this manuscript:

AVM	intracranial arteriovenous malformation
CT	Computed Tomography
MCTA	multiphase CT angiography
